# *Trp* RNA-Binding Attenuation Protein: Modifying Symmetry and Stability of a Circular Oligomer

**DOI:** 10.1371/journal.pone.0044309

**Published:** 2012-09-06

**Authors:** Oliver W. Bayfield, Chao-Sheng Chen, Andrea R. Patterson, Weisha Luan, Callum Smits, Paul Gollnick, Alfred A. Antson

**Affiliations:** 1 York Structural Biology Laboratory, Department of Chemistry, University of York, York, United Kingdom; 2 Department of Biological Sciences, State University of New York, Buffalo, New York, United States of America; Saint Louis University, United States of America

## Abstract

**Background:**

Subunit number is amongst the most important structural parameters that determine size, symmetry and geometry of a circular protein oligomer. The L-tryptophan biosynthesis regulator, TRAP, present in several *Bacilli*, is a good model system for investigating determinants of the oligomeric state. A short segment of C-terminal residues defines whether TRAP forms an 11-mer or 12-mer assembly. To understand which oligomeric state is more stable, we examine the stability of several wild type and mutant TRAP proteins.

**Methodology/Principal Findings:**

Among the wild type *B. stearothermophilus*, *B. halodurans* and *B. subtilis* TRAP, we find that the former is the most stable whilst the latter is the least. Thermal stability of all TRAP is shown to increase with L-tryptophan concentration. We also find that mutant TRAP molecules that are truncated at the C-terminus - and hence induced to form 12-mers, distinct from their 11-mer wild type counterparts - have increased melting temperatures. We show that the same effect can be achieved by a point mutation S72N at a subunit interface, which leads to exclusion of C-terminal residues from the interface. Our findings are supported by dye-based scanning fluorimetry, CD spectroscopy, and by crystal structure and mass spectrometry analysis of the *B. subtilis* S72N TRAP.

**Conclusions/Significance:**

We conclude that the oligomeric state of a circular protein can be changed by introducing a point mutation at a subunit interface. Exclusion (or deletion) of the C-terminus from the subunit interface has a major impact on properties of TRAP oligomers, making them more stable, and we argue that the cause of these changes is the altered oligomeric state. The more stable TRAP oligomers could be used in potential applications of TRAP in bionanotechnology.

## Introduction

Cyclic protein oligomers pose promise as the basis for engineering molecular machines, including those that operate by rotation of one or more components about a principal axis. Their suitability, owing to their unique geometry, is evident by their frequent occurrence in key bio-mechanical and regulatory roles. Examples include circular protein oligomers found in flagellar motors [1], the ATP synthase [2] and the molecular motor of tailed bacteriophages which translocate DNA into the capsid during viral particle assembly [3]–[5]. The stoichiometry of the MS-ring and C-ring in flagellar motors are variable [6]. Likewise, the central component of the bacteriophage DNA-translocating motor can also exist in different oligomeric states [5]. This demonstrates a general problem associated with instability of circular oligomers and variation in their oligomeric states when they are extracted from their natural environment. Engineering stable molecular devices of suitable size, geometry, and hence subunit number is of interest for applications in molecular biology and medicine. For example, applications of viral DNA-translocating devices in the transfer of genetic information across biological membranes are attractive. Recent studies aimed at the incorporation of a portal protein into lipid bilayers look particularly promising [7].

The tryptophan RNA-binding attenuation protein, TRAP, possesses similar geometric features to other circular assemblies, being assembled of multiple subunits and containing a central tunnel. Typically composed of 11 or 12 identical subunits, with a molecular weight of 90.6 to 101.9 kDa [8], [9], TRAP regulates L-tryptophan biosynthesis genes by attenuation in many *Bacilli* [10]. When activated by increased levels of L-tryptophan – bound to receptor sites nestled between adjacent subunits – TRAP binds single stranded RNA at the leader region of the *trpEDCFBA* operon transcript. This modulates terminator hairpin formation and so downregulates L-tryptophan biosynthesis [8]. In this study TRAP is employed as a model for investigating the influences of oligomeric state and stability of such assemblies. To date TRAP has already alluded to its value in bionanotechnology, showing that mutations to residues protruding into the central cavity allow gold nanoparticle binding [11]. Nanotubes consisting of stacked TRAP rings linked by disulfide bonds have also been shown to self-assemble, in response to the introduction of cysteine residues on the ring-faces orthogonal to the principal axis [12].

TRAP oligomers have previously been reported to be highly thermostable [13]. Predictably, we find that the melting temperature is highest in the case of TRAP from *B. stearothermohphilus*, a hyperthermophile. The presence of L-tryptophan – and hence the bound holo-TRAP state – has been associated with a greater structural rigidity and integrity [14]. Prior to the recent determination of the *B. stearothermophilus* apo-TRAP crystal structure [15] TRAP was crystallized in the presence of L-tryptophan [8], [9]. We find that L-tryptophan binds with a K_d_ in the 2.8 µM to 6.9 µM range, with small variation between TRAP from different species. We explore how the presence of L-tryptophan and its’ binding affects thermal stability in the context of the three wild type and three mutant TRAP molecules. We also explore the influence of the oligomeric state on thermal stability by comparing wild type 11-subunit and mutant 12-subunit molecules from the same species. Finally, we show how the 11-subunit to 12-subunit switch could be induced by the point mutation S72N. The analysis includes crystal structure and native mass spectrometry data on *B. subtilis* S72N TRAP. We find that the increase in subunit number increases the stability of the assembly.

## Results

### Crystal Structure and Native Mass Spectrometry of *B. subtilis* S72N TRAP

**Figure 1:**
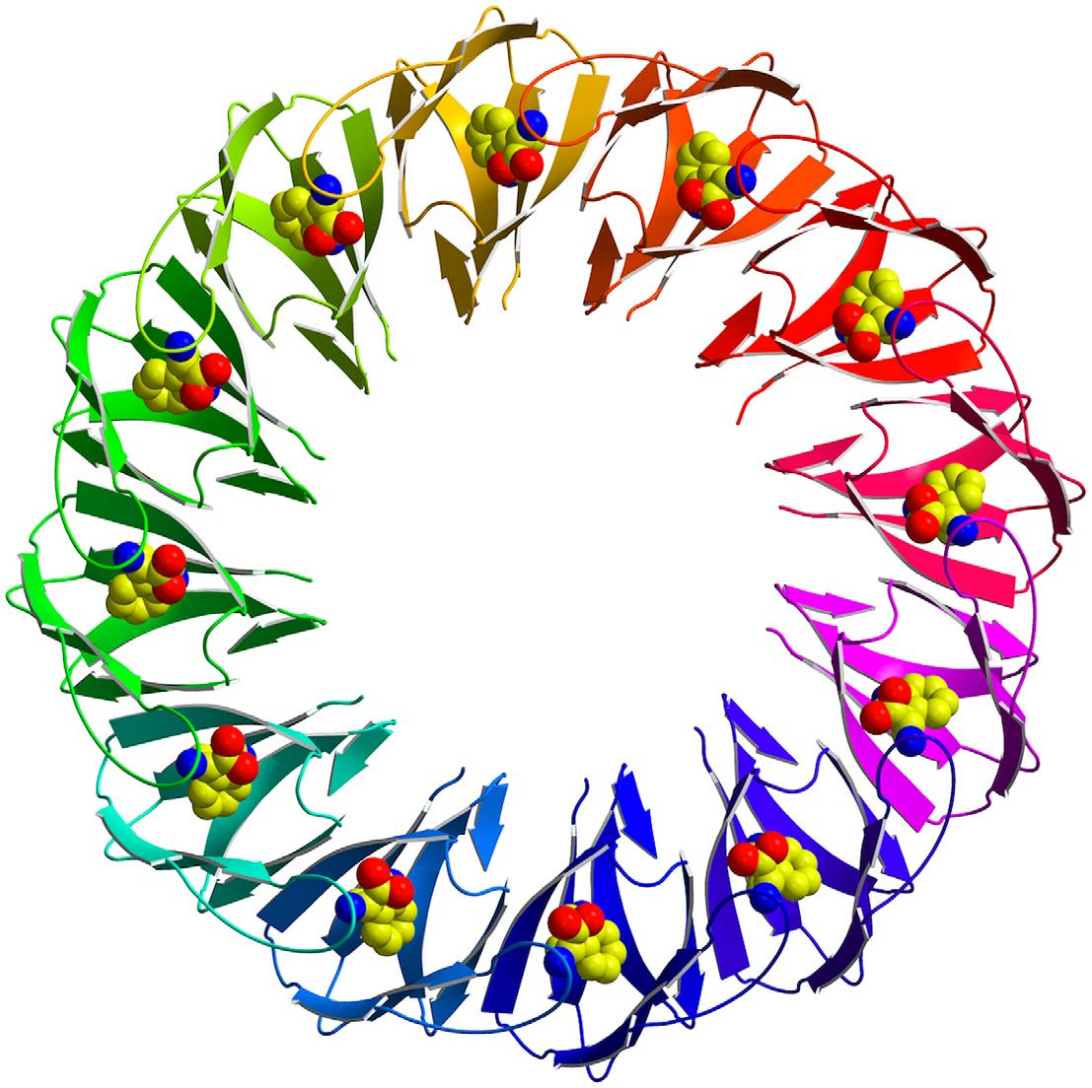
Ribbon diagram of the *B. subtilis* S72N TRAP viewed along the 12-fold axis. Each subunit is shown in a different colour. L-tryptophan molecules are shown as van der Waals models with oxygen atoms in red, nitrogen atoms in blue and carbon atoms in yellow.

**Figure 2:**
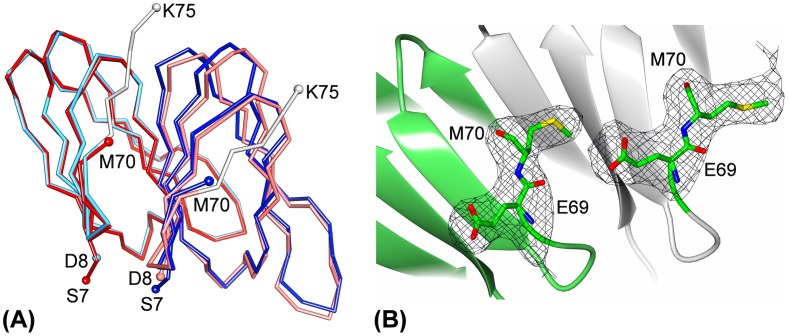
Structural differences between 11-subunit and 12-subunit TRAP. (**A**) Comparison of the *B. subtilis* wild type (red) and S72N mutant TRAP (blue). Two neighboring subunits were least-square fitted using main chain atoms of the subunit on the left. The C-terminal residues that are pivoted out of the subunit interface in S72N TRAP are highlighted in white on the wild type TRAP, starting from residue 69. (**B**) C-terminal residues E69 and M70 are shown in sticks with main chain in yellow and side chains in turquoise, the rest of each subunit is shown in ribbons. The weighted *F*_o_ – *F*_c_ omit maps, contoured at 2σ, were calculated after omitting residues 69 and 70 from the final model and 10 cycles of refinement.

We previously demonstrated the importance of the C-terminus in oligomer formation during *B. subtilis* TRAP assembly [9]. However, this work required deletion of the C-terminus to increase the subunit stoichiometry and we questioned if a more subtle mutation at the C-terminus could induce formation of a 12-subunit assembly. Based on the previous structural work, we chose the S72N mutation because we expected that the bulky side chain at this position would result in the exclusion of N72 and following C-terminal residues from the subunit interface. The crystal structure of *B. subtilis* S72N TRAP, determined at 2.7 Å, reveals a circular 12-mer assembly (**Figure 1**), which is similar to previously reported crystal structures of TRAP. In the crystal the 12 subunit assembly is generated by combination of six TRAP subunits, present in the asymmetric unit, with the crystallographic 2-fold axis of the P2_1_2_1_2 space group (**Table 1**). The final electron density maps allowed positioning of all residues, with the exception of the four N-terminal residues and the five C-terminal residues of most subunits, for which no clear electron density has been observed, indicating their flexibility. The structure of the S72N TRAP was compared against the wild type TRAP (**Figure 2A**), which highlighted change in the conformation of the C-terminus in the mutant protein, starting from position 69. The weighted *F_o_ – F_c_* omit electron density maps for residues E69 and M70 (**Figure 2B**) demonstrate the absence of interpretable electron density beyond position 70 in all chains, indicating that five C-terminal residues are disordered. The crystal structure compares well with our predictions on the effect of the point mutation, in inducing the removal of the C-terminus from the subunit interface through steric hindrance, thus facilitating rigid-body rotations of subunits for the accommodation of one additional subunit into the ring.

**Table 1:** Data collection and refinement statistics for *B. subtilis* S72N TRAP.

Data collection	
Space group	P2_1_2_1_2
Unit cell	*a* = 109.4 Å, *b* = 110.2 Å, *c* = 46.2 Å
Resolution	25–2.7 Å (2.80–2.70 Å)
No. of reflections	14253 (1025)
Redundancy	6.3 (4.2)
*R*_merge_ a, %	9.7 (50.2)
Completeness, %	91.9 (68.2)
I/σ	15.3 (2.8)
Wilson B factor	86.1
**Refinement**	
Resolution range	25–2.72 Å (2.79–2.72 Å)
No. of reflections used in refinement	13396
No. of reflections excluded from refinement	825
*R* factor^b^, %	20.9 (34.1)
Free *R* factor^b^, %	24.5 (38.2)
**Number of atoms**	
Protein	2952
Ligand	90
Water molecules	27
Average B factor (protein)	83.8
Average B factor (solvent)	82.5
Rmsd bond lengths	0.009 Å
Rmsd bond angles	1.2°
**Ramachandran plot**	
Most favored regions, %	97.8
Additionally allowed regions, %	2.2

Values in parentheses are for the highest resolution shell.

a *R_merge_*  =  ∑*_hkl_*∑_i_|*I_i_(h)* - <*I(h)>*|/∑*_hkl_*∑_i_ *I_i_(h)*, where *I(h)* is intensity of reflection *h*, <*I(h)>* is average value of intensity, the sum ∑*_hkl_* is over all measured reflections and the sum ∑_i_ is over *i* measurements of a reflection.

b Crystallographic *R  = * ∑*_hkl_*||*F_obs_* - *F_calc_*||/∑*_hkl_*|*F_obs_|*, *R_free_* was calculated using a randomly chosen set of reflections that were excluded from the refinement.

To understand if the 12-mer state of the S72N TRAP observed in the X-ray structure, predominates in solution, we analyzed this protein by native mass spectrometry (**Text S1**). The 12-mer state was identified as the dominant species in the mass spectra (**Figure S1**), as opposed to the wild type protein which forms 11-mers [16].

### 12-mer TRAP Assemblies are More Stable

**Figure 3:**
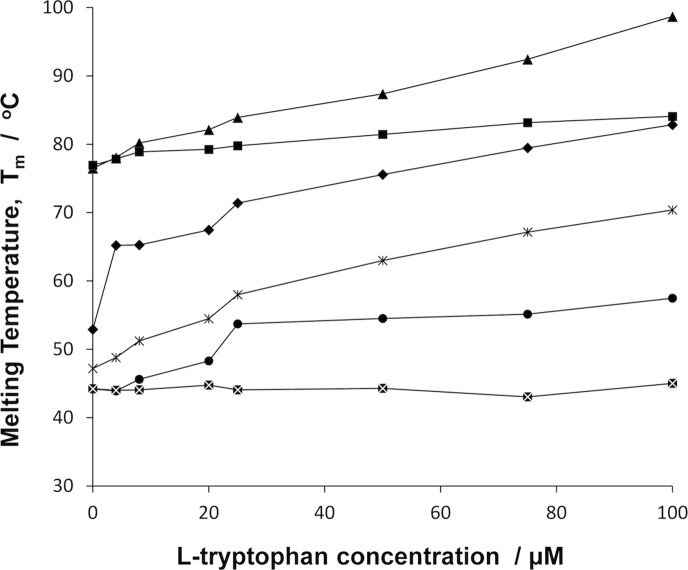
Melting temperatures of different TRAP oligomers. The dependence of melting temperature is shown for six TRAP oligomers, as a function of L-tryptophan concentration, determined by dye-based scanning fluorimetry. From the top: *B. stearothermophilus* E71stop 12-mer (triangle), *B. stearothermophilus* wild type 11-mer (square), *B. halodurans* wild type 12-mer (diamond), *B. subtilis* K71stop 12-mer (open cross), *B. subtilis* S72N 12-mer (circle), *B. subtilis* wild type 11-mer (crossed square).

Melting temperatures for six TRAP molecules, including three wild type and three mutant TRAP listed in Table 2, were determined at varied L-tryptophan concentrations (0–100 µM) using dye-based scanning fluorimetry as described in the methods. Fluorescence versus temperature melting curve data (**Figure S2, S3**) was averaged between four observations and the midpoints of fitted sigmoidal curves were taken as the melting temperatures, T_m_. Melting temperatures (**Table S1**) were then plotted against L-tryptophan concentration (**Figure 3**).

**Table 2:** L-tryptophan binding constants (K_d_) of three wild type and three mutant TRAP.

TRAP oligomer	Number of subunits	K_d_/µM
*B. halodurans* wild type	12	4.5
*B. stearothermophilus* wild type	11	3.2
*B. stearothermophilus* E71stop	12	3.9
*B. subtilis* wild type	11	6.9
*B. subtilis* K71stop	12	6.5
*B. subtilis* S72N	12	2.8

As the fluorescent dye could potentially bind in the tryptophan-binding pocket, we employed CD spectroscopy (**Text S2**) to verify that melting temperatures were not perturbed by interaction with the dye, **Figure S2**. We note that there is coherence between melting temperatures derived from the two respective techniques.

Increasing the L-tryptophan concentration increases the thermal stability of all the TRAP proteins except for wild type *B. subtilis* TRAP. *B. halodurans* TRAP was included as a reference, as it is naturally a 12-mer, without engineering. Throughout the L-tryptophan concentrations for which the T_m_ was measured, the wild type *B. subilis* TRAP was least stable, while the *B. stearothermophilus* was most stable. Significantly, the mutant 12 subunit assemblies were at least as stable, but usually more stable than the 11 subunit wild-type, at all L-tryptophan concentrations measured. This stabilization was particularly marked at high L-tryptophan concentrations as the mutant 12-mer subunit TRAPs have a larger increase in T_m_ with increasing L-tryptophan concentration than their wild type 11-mer counterparts. For example, from 0 to 100 µM L-tryptophan, *B. subtilis* wild type increases in stability by 0.8°C, while the T_m_ of the K71stop and S72N 12-mer mutants increases by 23.2°C and 13.3°C respectively. The *B. stearothermophilus* wild type TRAP increases in stability by 7.1°C, whereas the T_m_ of E71stop TRAP increases by 20.2°C. The thermal stability of *B. subtilis* wild type TRAP appeared to show little dependence on L-tryptophan concentration up to 100 µM. We observed that the stability of the wild type 12-mer *B. halodurans* TRAP lies midway between that of mutant 12-mers, *B. subtilis* K71stop and *B. stearothermophilus* E71stop TRAP (**Figure 3**).

### L-Tryptophan Binding

The increased stabilization by L-tryptophan of the 12-subunit assemblies, as compared to 11-mers, was unexpected. To investigate this further we measured the affinity of each TRAP for L-tryptophan (**Table 2**). A comparison of the dissociation constants of the mutant 12-mers to their 11-mer wild type counterparts showed no obvious correlation. Although an 11-mer to 12-mer transition in *B. subtilis* TRAP increases the affinity of binding, this is not observed in *B. stearothermophilus* TRAP. This suggests stabilization by L-tryptophan is not directly related to affinity towards L-tryptophan.

## Discussion

The conformation of the C-terminus has previously been shown to play a major role in determining whether the 11-mer or the 12-mer oligomeric state assembles [9], and the new structure of the S72N mutant reinforces this. The relationship between subunit number and thermal stability has for the first time been probed, revealing the 12-mer configuration is more stable than the 11-mer counterpart.

### Comparison of Thermal Stability in Wild Type TRAP

We compared the thermal stability of *B. stearothermophilus*, *B. halodurans*, and *B. subtilis* wild type TRAP. The relative thermal stabilities of the oligomers reflect the temperature environments of the respective species’ habitats, as might be expected. For example, at 50 µM L-tryptophan, the melting temperatures for the respective wild type TRAP are: *B. stearothermophilus*, 81.4°C; *B. halodurans*, 75.6°C; *B. subtilis*, 44.3°C. These melting temperatures reflect the respective species’ hyperthermophilic, thermophilic and mesophilic origins respectively. This relationship appears to hold across all of the L-tryptophan concentration range (**Figure 3**).

Within these wild type TRAP oligomers, *B. stearothermophilus* and *B. subtilis* both have 11-mer TRAP, and *B. halodurans* has 12-mer TRAP. It is apparent therefore that the temperature environment in which the species have evolved is the overriding factor in determining the thermal stability of the wild type TRAP, as we have an 11-mer as both the most and least stable. However, we find that one effect of inducing a change of oligomeric state from 11-mer to 12-mer by mutations is increased thermal stability, implying that species-specific structural characteristics unrelated to subunit number are not the only determinant of thermal stability.

### C-terminal Truncation Induces 11- to 12-mer Transition and Increases Stability

Comparing the structure of the 12-mer *B. halodurans* TRAP with that of the 11-mer structures of *B. subtilis* TRAP and *B. stearothermophilus* TRAP, the importance of the C-terminus (residues 71 to 75) becomes apparent. In *B. halodurans* TRAP, the nature of the last four amino acids dictate that the C-terminal segment is excluded from the subunit-subunit interface, thereby generating favorable interactions at the outer surface of the oligomer. This is considered as the crucial factor in permitting the higher oligomeric state, which is related to the 11-mer state by a 2.7^o^ rigid-body rotation of adjacent subunits [9]. Hence what can be considered macroscopically as the result of the extraction of a thin “wedge” between subunits on the outer side of the ring, has the effect of decreasing the angle that a single subunit contributes towards the full 360°.

Taking our investigation a step further, we focused further studies at determining whether there is a general relationship between the oligomeric state of TRAP and its thermal stability, hence exploring factors that are not species-specific. The scenario found in the 12-mer *B. halodurans* TRAP, regarding the exclusion of the C-terminus, could logically be mimicked by truncating the 4 or 5 C-terminal residues, removing this segment from the subunit interface. This is observed in the previously determined structures of *B. stearothermophilus* E71stop TRAP and *B. subtilis* K71stop TRAP [9]. With respect to thermal stability accompanying the 11-mer to 12-mer transition, we find that this truncation results in an increase in melting temperature, so that at 50 µM L-tryptophan the increase in melting temperature between *B. stearothermophilus* wild type and E71stop is 6°C, from 81.4°C to 87.4°C. The equivalent increase between *B. subtilis* wild type and K71stop is 18.7°C, 44.3°C to 63.0°C. This hints at an increase in thermal stability in response to incorporation of an additional subunit into the ring. However, this difference in melting temperature could also be a result of the absence of the C-terminal residues themselves. For instance, we note that both *B. subtilis* and *B. stearothermophilus* wild type TRAP contain two flexible lysine residues at their C-termini [17] that can contribute electrostatically to the overall stability of TRAP. To understand which of these two factors – C-terminus or oligomeric state – has the greater influence we compared thermal stability of wild type *B.* subtilis TRAP 11-mer with that of the *B. subtilis* S72N TRAP 12-mer, which still possesses the C-terminal segment but has it excluded from the subunit interface for steric reasons.

### S72N Mutation also Increases Subunit Number and Stability

Exclusion of the C-terminus from the subunit interface can be achieved by introducing steric strain at a key region towards the C-terminus that causes the segment to pivot out. In this respect, exclusion of the C-terminus in S72N is achieved in a manner more similar to how exclusion is achieved in *B. halodurans* TRAP. Residue 72 in particular (Ser 72 in *B. subtilis* TRAP) – which sits at the subunit-subunit interface in both wild type 11-mer structures studied – serves as a critical determinant in the conformation of the C-terminus. This is evident from the crystal structure presented in this study. From the structure of the S72N TRAP we see that substituting serine 72 for a bulkier amino acid, asparagine, does indeed introduce the necessary constraints to exclude the subsequent segment from the subunit interface. The thermal stability of this mutant 12-mer TRAP, S72N, is higher than that of its wild type 11-mer counterpart. For example at 50 µM L-tryptophan the melting temperature of the wild type and mutant oligomers are 44.°C and 54.5°C respectively, yielding a stabilization of 10.2°C. This is significant, albeit it is less compared to the 18.7°C stabilization we see from C-terminal truncation (i.e. *B. subtilis* K71stop).

We conclude that by inducing an expansion in the oligomeric state from 11 to 12 subunits the thermal stability of the oligomer is increased. This increase is predominantly a result of the oligomeric state transition, and to a much lesser extent the influence of C-terminal or other specific residues. A potential, general relationship in TRAP oligomers therefore exists, in which melting temperature depends on subunit number. Further studies should be focused on evaluation of influence of the oligomeric state of TRAP on its activity *in vivo*.

### Binding Affinity Towards L-tryptophan

To explore whether the stabilization by L-tryptophan was directly related to the affinity of TRAP oligomers to the binding of L-tryptophan, dissociation constants were measured. Results showed no obvious correlation with thermal stability or subunit number (**Table 2**). Although between *B. subtilis* wild type and S72N mutant TRAP, the 11-mer to 12-mer transition does coincide with a slight increase in affinity for L-tryptophan, these changes are only twofold, implying there are other more significant influences.

### Increasing L-tryptophan Concentration Increases Thermal Stability

For most TRAP oligomers studied we observe an increase in melting temperature in response to an increase in L-tryptophan concentration from 0 µM to 100 µM added. The trend does not appear in *B. subtilis* wild type TRAP however, over the L-tryptophan concentration range studied. Most concentrations in the monitored range represent many times the typical K_d_ value, hence L-tryptophan binding-sites in TRAP are expected to be saturated. This therefore suggests a degree of non-specific binding of L-tryptophan to TRAP as the principal cause of the stabilization, over a thermodynamic or kinetic influence involving the L-tryptophan binding sites. It is likely that non-specific binding, beyond the 1∶1 stoichiometry, would hence increase with increasing concentration of L-tryptophan.

### Conclusions

This study demonstrates how the oligomeric state of a circular protein can be changed by introducing a point mutation at a subunit interface. Importantly, we demonstrate that a change in subunit number changes the stability of a circular assembly *in vitro*. In the case of TRAP, used in our studies, an increase in subunit number increases the stability for the oligomeric assembly. Predictably, this stability is dependent on solvent conditions, specifically L-tryptophan concentration. We conclude that a similarly subtle approach could be used during protein engineering of other ring-like structures, for stabilizing a particular oligomeric state thereby modifying dimensions, symmetry and stability.

## Materials and Methods

### Gene Cloning, Protein Purification, Crystallization and Data Collection

The *B. subtilis* S72N mutant TRAP was generated using the QuikChange kit (Stratagene, US) and a pET9a plasmid containing the wild type gene. S72N TRAP proteins were produced and purified as described previously [18]. Before crystallization, protein samples were transferred into solution containing 2 mM Tris (pH 8.5), 300 mM NaCl and purified by size-exclusion chromatography using Superdex 200 Column (GE healthcare, UK). TRAP samples were initially analysed by size-exclusion chromatography, employing a Superdex 200 10/300 column (GE healthcare, UK) for the preliminary oligomeric assembly analysis. A 200–300 µg TRAP sample was injected and eluted by a buffer containing 20 mM Tris-HCl, 30 mM NaCl, and 0.1 mM L-tryptophan (pH 8.5). TRAP samples with an elution volume less than 14.5 ml were selected for further analysis by native mass spectrometry analysis (**Figure S1**) and X-ray crystallographic studies.

Crystallization was carried out at 18°C by hanging drop vapor diffusion. For crystallization, *B. subtilis* S72N TRAP was transferred into solution containing 10 mM triethanolamine (pH 8.0), 100 mM NaCl, 15 mM L-tryptophan and concentrated to 33.5 mg/ml. The reservoir contained 100 mM Bis-Tris propane (pH 8.5), 200 mM KSCN and 13% PEG 3350 (v/v). Protein crystals were frozen using solutions containing all the crystallization ingredients with addition of 20% glycerol (v/v). The X-ray data were collected at 120 K at I04 beamline at the Diamond Light Source. Data were processed using HKL2000 [19], (**Table 1**).

### Structure Determination and Refinement

All crystallographic calculations were carried out using the CCP4 program package [20]. The structure was determined by molecular replacement using MOLREP [21] with three adjacent subunits of *B. stearothermophilus* TRAP as a search model. Refinement was performed by REFMAC [22] and model rebuilding was done using COOT [23]. Water molecules were added automatically with the program ARP/wARP [24] and further corrected using maximum likelihood-weighted 2|F_o_| - |F_c_| and |F_o_| - |F_c_| electron density maps. Molecular contacts between adjacent monomers of TRAP were examined by CONTACT [20]. **Figure 1** was generated using Bobscript [25], **Figure 2A** using UCSF Chimera [26], and **Figure 2B** using CCP4 mg [27].

### Dye-based Scanning Fluorimetry

Dye-based scanning fluorimetry, or the Thermofluor method, was performed as previously described [28], [29]. Protein samples were prepared by transferring to 50 mM Tris pH 8, 150 mM NaCl, 10 mM MgCl_2_, with variable L-tryptophan concentration, in 30 kDa cut-off Vivaspin concentrators, and diluting to 1 mg/ml, monitoring concentration by the Comassie-blue Bradford method. L-tryptophan concentration was varied from 0 to 0.1 mM (final well concentration). Samples were transferred to 96-well thin-walled clear PCR plates. Each well contained 30 µl of solution, consisting of 15 µl of the 1 mg/ml protein (yielding a final concentration of 0.5 mg/ml), 1/1000 diluted SYPRO Orange (Sigma-Aldrich) and 50 mM Tris pH 8 with a specified L-tryptophan concentration. A Stratagene Mx3005P QPCR instrument was programmed to increase temperature in 1°C increments at 30 second intervals, from 25°C to 95°C with 470 nm excitation, measuring fluorescence at 570 nm.

### Measurement of L-tryptophan Dissociation Constants

L-tryptophan binding to TRAP was measured using a fluorescence assay based on competition between L-tryptophan and 1-anilionaphthalene-8-sulfonic acid (ANS) as described previously [30]. ANS fluoresces weakly in solution but fluoresces strongly at 460 nm with 372 nm excitation upon binding to TRAP. L-tryptophan has a greater affinity than ANS for TRAP, and displaces ANS thus reducing the fluorescence. The change in fluorescence, ΔF, was monitored using a LS-50B fluorometer (Perkin Elmer). A 100 µL sample of 15 µM TRAP was prepared in 60 µM ANS and 0.5 mM sodium phosphate at pH 8.0. Readings of fluorescence intensity were taken from 0 to 2000 µM tryptophan. At each concentration of tryptophan, fluorescence intensity was collected at room temperature for one minute to allow the reaction to reach equilibrium. Fluorescence intensity was averaged for each concentration of L-tryptophan and then used to calculate the change in fluorescence (ΔF). The maximal change in fluorescence was set to 100% and values of ΔF were normalised accordingly. GraphPad Prizm 4 was used to plot % ΔF as a function of tryptophan concentration to obtain L-tryptophan dissociation constants and binding curves.

### Data Deposition

Refined coordinates of the *B. subtilis* S72N TRAP and structure factors have been deposited with the Protein Data Bank under accession code 4B27.

## Supporting Information

Figure S1 **Native mass (Nanoflow electrospray) spectrum of** ***B. subtilis*** **S72N TRAP.** m/z corresponds to the mass-to-charge ratio. Red hexagons correspond to 12-mer TRAP species without bound tryptophan.
(DOCX)

Figure S2
**Melting curves of** ***B. halodurans*** **and** ***B. stearothermophilus*** **wild type TRAP.** Normalised fluorescence data (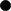) are overlaid with CD data recorded at 205 nm (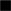), both at 50 µM L-tryptophan in the sample. (**A**) *B. halodurans* wild type TRAP (12-mer). (**B**) *B. stearothermophilus* wild type TRAP (11-mer). Sigmoidal curves were fitted to CD spectroscopy data (red) for comparison against fluorescence data. The melting temperatures represented by the midpoints of these curves were found to be 78.5°C and 81.6°C for (A) and (B) respectively. These compare well with the melting temperatures derived from curves fitted to the fluorescence data, 75.6°C and 81.4°C respectively (Table S1).(DOCX)

Figure S3 (**A**) Overlays of 8 normalised fluorescence versus temperature curves of *B. stearothermophilus* E71Stop TRAP, with concentration of L-tryptophan increasing left to right with lightening shades of green: 0, 4, 8, 20, 25, 50, 75, 100 µM. (**B**)****Curves derived from four replicates at two concentrations of L-tryptophan, for *B. stearothermophilus* E71Stop TRAP: 0 µM L-tryptophan (dark red = mean; pink = replicates), 50 µM L-tryptophan (dark blue = mean; light blue = replicates), normalised to the mean curve, showing equivalent midpoint and plateau positions within replicates.
(DOCX)

Table S1 **Melting temperatures for TRAP oligomers over 0 to 100 mM L-tryptophan concentration.** *B. halo.* (*B. halodurans*); *B. stearo.* (*B.stearothermophilus*); *B. sub.* (*B. subtilis*); WT (wild type). Standard deviations across four repeated measurements are shown in the parentheses.
(DOCX)

Text S1 **Materials and Methods - Native Mass Spectrometry.**
(DOCX)

Text S2 **Materials and Methods - Circular Dichroism spectroscopy.**
(DOCX)
