# Supplementary figures and images for: Trp RNA-Binding Attenuation Protein: Modifying Symmetry and Stability of a Circular Oligomer
Source: PLoS One. 2012 Sep 6;7(9):e44309. doi: 10.1371/journal.pone.0044309 (PMC3435397; doi:10.1371/journal.pone.0044309)

**Figure S1.**

**
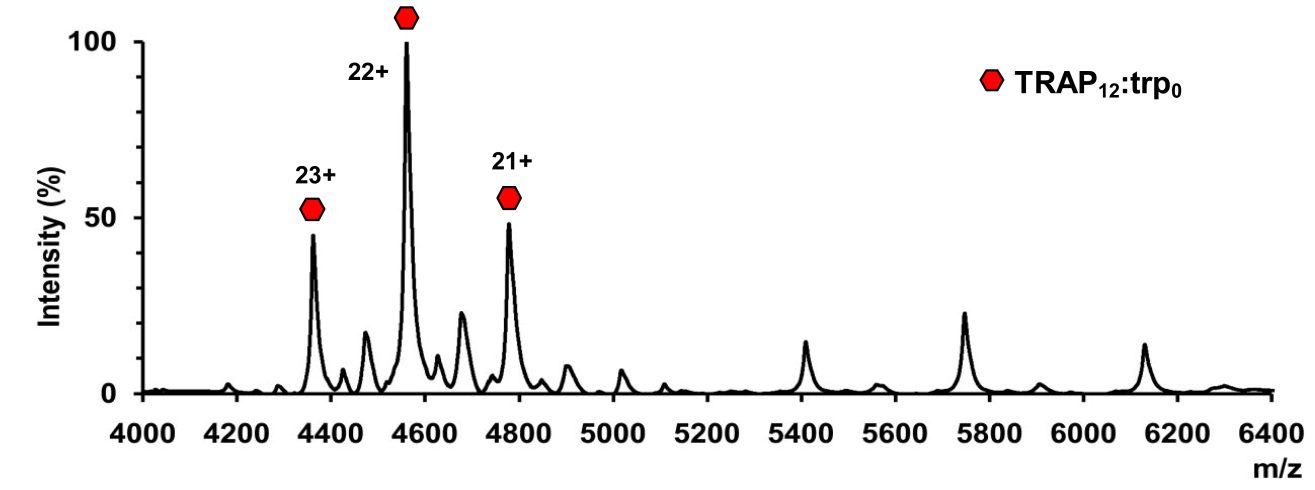
**

Supplement: Figure S1 — Native mass (Nanoflow electrospray) spectrum of B. subtilis S72N TRAP. m/z corresponds to the mass-to-charge ratio. Red hexagons correspond to 12-mer TRAP species without bound tryptophan. (DOCX) [file pone.0044309.s001.docx]

**Figure S2.**

**
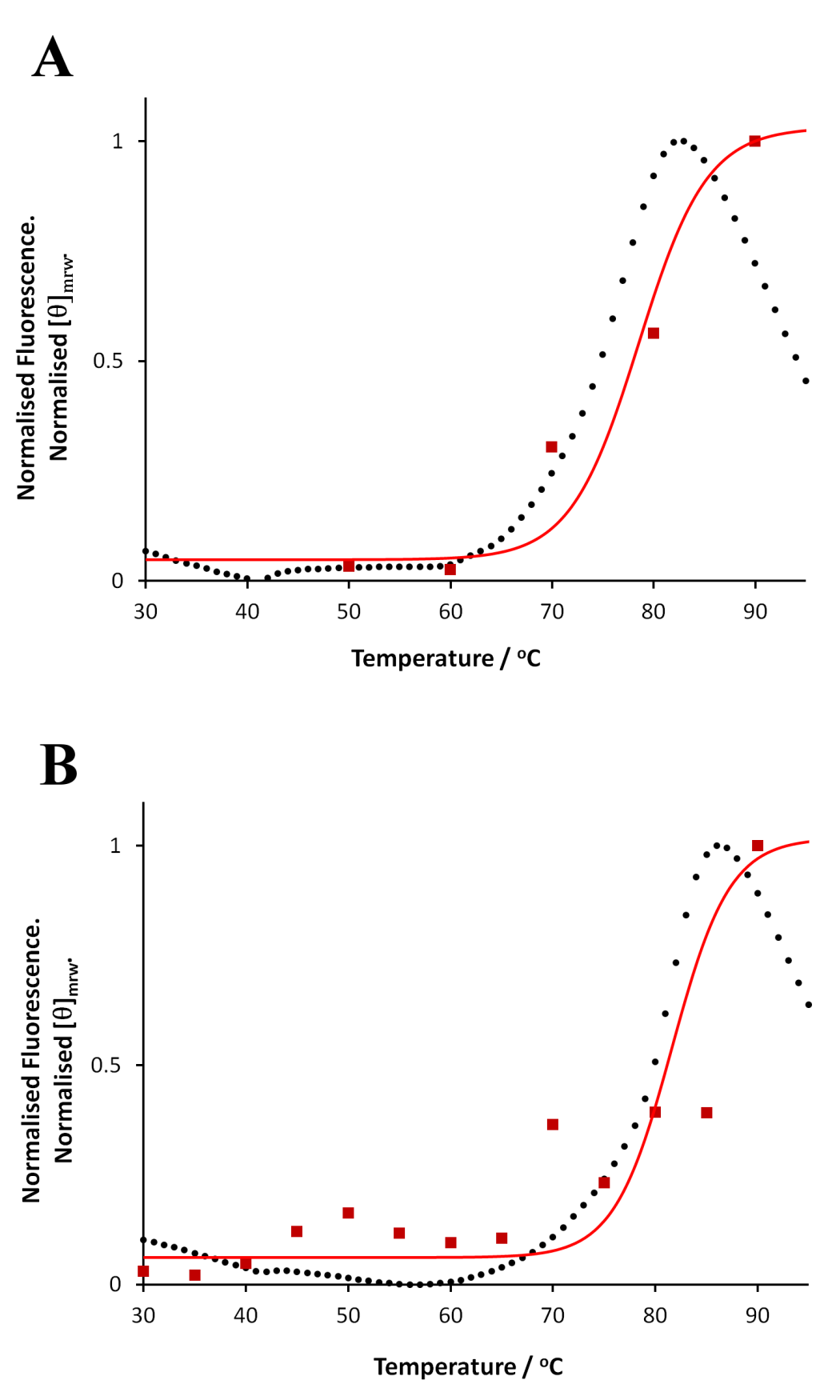
**

Supplement: Figure S2 — Melting curves of B. halodurans and B. stearothermophilus wild type TRAP. Normalised fluorescence data () are overlaid with CD data recorded at 205 nm (), both at 50 µM L-tryptophan in the sample. (A) B. halodurans wild type TRAP (12-mer). (B) B. stearothermophilus wild type TRAP (11-mer). Sigmoidal curves were fitted to CD spectroscopy data (red) for comparison against fluorescence data. The melting temperatures represented by the midpoints of these curves were found to be 78.5°C and 81.6°C for (A) and (B) respectively. These compare well with the melting temperatures derived from curves fitted to the fluorescence data, 75.6°C and 81.4°C respectively (Table S1). (DOCX) [file pone.0044309.s002.docx]

**Figure S3.**

**
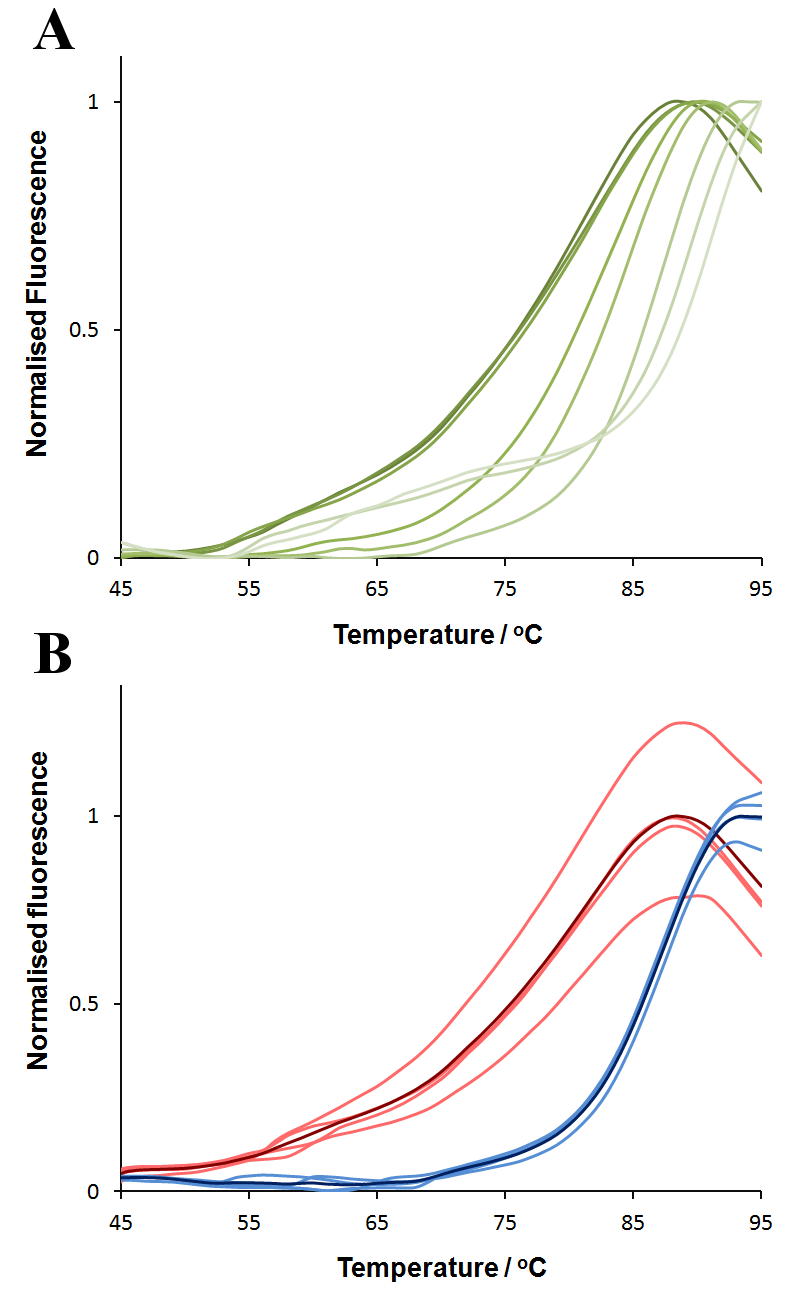
**

Supplement: Figure S3 — (A) Overlays of 8 normalised fluorescence versus temperature curves of B. stearothermophilus E71Stop TRAP, with concentration of L-tryptophan increasing left to right with lightening shades of green: 0, 4, 8, 20, 25, 50, 75, 100 µM. (B)Curves derived from four replicates at two concentrations of L-tryptophan, for B. stearothermophilus E71Stop TRAP: 0 µM L-tryptophan (dark red = mean; pink = replicates), 50 µM L-tryptophan (dark blue = mean; light blue = replicates), normalised to the mean curve, showing equivalent midpoint and plateau positions within replicates. (DOCX) [file pone.0044309.s003.docx]
