# Supplementary material for: Trp RNA-Binding Attenuation Protein: Modifying Symmetry and Stability of a Circular Oligomer
Source: PLoS One. 2012 Sep 6;7(9):e44309. doi: 10.1371/journal.pone.0044309 (PMC3435397; doi:10.1371/journal.pone.0044309)
Supplement: Table S1 — Melting temperatures for TRAP oligomers over 0 to 100 mM L-tryptophan concentration. B. halo. (B. halodurans); B. stearo. (B.stearothermophilus); B. sub. (B. subtilis); WT (wild type). Standard deviations across four repeated measurements are shown in the parentheses. (DOCX) [file pone.0044309.s004.docx]

**Table S1.**

| **L-trp conc. / mM** | ***B. halo.* WT** | ***B. stearo.* WT** | ***B. stearo.* E71Stop** | ***B. sub.* WT** | ***B. sub.* K71Stop** | ***B. sub.* S72N** |
| --- | --- | --- | --- | --- | --- | --- |
| **0** | 52.9 (0.51) | 76.9 (0.16) | 76.5 (0.53) | 44.2 (0.10) | 47.2 (0.55) | 44.2 (1.77) |
| **4** | 65.2 (1.43) | 77.8 (0.33) | 78.1 (0.41) | 44.0 (1.68) | 48.8 (0.51) | 43.9 (2.03) |
| **8** | 65.3 (1.11) | 78.9 (0.23) | 80.2 (1.39) | 44.1 (1.30) | 51.2 (0.81) | 45.6 (1.48) |
| **20** | 67.5 (0.77) | 79.3 (0.49) | 82.1 (0.56) | 44.8 (0.12) | 54.5 (0.98) | 48.3 (2.12) |
| **25** | 71.4 (0.23) | 79.8 (0.61) | 83.9 (0.20) | 44.1 (0.44) | 58.0 (0.31) | 53.7 (1.72) |
| **50** | 75.6 (1.03) | 81.4 (0.23) | 87.4 (0.33) | 44.3 (1.51) | 63.0 (0.41) | 54.5 (1.61) |
| **75** | 79.5 (0.51) | 83.2 (0.64) | 92.4 (0.68) | 43.0 (0.77) | 67.1 (0.62) | 55.1 (1.35) |
| **100** | 82.9 (0.20) | 84.1 (0.25) | 98.7 (1.24) | 45.0 (1.62) | 70.4 (0.36) | 57.5 (2.41) |
