# Supplementary material for: Trp RNA-Binding Attenuation Protein: Modifying Symmetry and Stability of a Circular Oligomer
Source: PLoS One. 2012 Sep 6;7(9):e44309. doi: 10.1371/journal.pone.0044309 (PMC3435397; doi:10.1371/journal.pone.0044309)
Supplement: Text S1 — Materials and Methods - Native Mass Spectrometry. (DOCX) [file pone.0044309.s005.docx]

**Text S1.** Materials and Methods - Native Mass Spectrometry.

Protein samples were in solution containing 100 mM ammonium acetate (pH 7.5) and 10 μM L-tryptophan at a concentration of 0.1 - 0.4 mg/ml. Mass spectrometry was performed using an orthogonal acceleration time-of-flight LCT premier XE system (Waters, MA, US), equipped with an offline nanoflow emitter (New Objective, MA, US). Mass spectra were acquired over the range 2000 to 8000 m/z, integrated over 5 sec intervals. Masslynx 4.1 software (Waters, MA, US) was employed to analyze the results. Molecular masses and standard deviations were calculated from the centroid values of species with at least three charge states. The data were calibrated externally with CsI solution (10 mg/ml).
