# Supplementary material for: Trp RNA-Binding Attenuation Protein: Modifying Symmetry and Stability of a Circular Oligomer
Source: PLoS One. 2012 Sep 6;7(9):e44309. doi: 10.1371/journal.pone.0044309 (PMC3435397; doi:10.1371/journal.pone.0044309)
Supplement: Text S2 — Materials and Methods - Circular Dichroism spectroscopy. (DOCX) [file pone.0044309.s006.docx]

**Text S2.** Materials and Methods - Circular Dichroism spectroscopy.

Samples were diluted to 1 mg/ml with 50 mM Tris pH 8, 150 mM NaCl, 10mM MgCl_2_, 0.050mM L-tryptophan, monitoring concentration by Bradford assay. 300 μl of sample was loaded into a quartz cuvette of path length 0.1 cm. To test interference of buffer components a blank was carried out at 30^o^C across the test wavelength range 185 nm to 260 nm. 1 mM and 0.5 mM L-tryptophan showed significant interference around 240 nm, whereas 0.05 mM (50 μM) showed little interference, thus allowing thermal unfolding to be monitored at these higher wavelengths. L-tryptophan (50 μM) was added owing to its presumed stabilising effect on TRAP. The strongest ellipticity signals for observing thermal unfolding of wild type TRAP appeared at 205nm, showing the greatest change in ellipticity with increasing temperature. Data presented in **Figure S2** was collected at 205 nm.

Conversion to mean residue molar ellipticity was performed using the equation:Θ

$$\left[ \Theta\right]_{\mathbf{mrw}}\mathbf{=}\frac{\Theta}{\mathbf{10}\boldsymbol{\cdot}\boldsymbol{n}\boldsymbol{\cdot}\boldsymbol{C}\boldsymbol{\cdot}\boldsymbol{l}}$$

**[**Θ**]_mrw_** is mean residue molar ellipticity / deg cm^2^ dmol^-1^.

Θ is ellipticity / mdeg.

***n*** is number of peptide bonds in the oligomer.

***C*** is concentration of the oligomer / mol dm^-3^.

***l*** is path length / cm.

Data were normalised following inversion on the y-axis to yield an analogous curve to that obtained by dye-based scanning fluorimetry for comparison.
